# Development of a novel murine delayed secondary fracture healing in vivo model using periosteal cauterization

**DOI:** 10.1007/s00402-019-03255-y

**Published:** 2019-08-09

**Authors:** Ina Gröngröft, Sandra Wissing, Dennis M. Meesters, Martijn Poeze, Romano Matthys-Mark, Keita Ito, Stephan Zeiter

**Affiliations:** 1 AO Research Institute Davos, Clavandelerstrasse 8, 7270 Davos Platz, Switzerland; 2 Department of Surgery and Trauma Surgery and NUTRIM School of Nutrition and Translational Research in Metabolism, Maastricht University Medical Center, Universiteitssingel 50, 6229 ER Maastricht, The Netherlands; 3 Department of Biomedical Engineering, Eindhoven University of Technology, Eindhoven, The Netherlands

**Keywords:** Fracture healing, Delayed union, Endochondral ossification, Mice

## Abstract

**Introduction:**

Delayed union and nonunion development remain a major clinical problematic complication during fracture healing, with partially unclear pathophysiology. Incidences range from 5 to 40% in high-risk patients, such as patients with periosteal damage. The periosteum is essential in adequate fracture healing, especially during soft callus formation. In this study, we hypothesize that inducing periosteal damage in a murine bone healing model will result in a novel delayed union model.

**Materials and methods:**

A mid-shaft femoral non-critically sized osteotomy was created in skeletally mature C57BL/6 mice and stabilized with a bridging plate. In half of the mice, a thin band of periosteum adjacent to the osteotomy was cauterized. Over 42 days of healing, radiographic, biomechanical, micro-computed tomography and histological analysis was performed to assess the degree of fracture healing.

**Results:**

Analysis showed complete secondary fracture healing in the control group without periosteal injury. Whereas the periosteal injury group demonstrated less than half as much maximum callus volume (*p* < 0.05) and bridging, recovery of stiffness and temporal expression of callus growth and remodelling was delayed by 7–15 days.

**Conclusion:**

This paper introduces a novel mouse model of delayed union without a critically sized defect and with standardized biomechanical conditions, which enables further investigation into the molecular biological, biomechanical, and biochemical processes involved in (delayed) fracture healing and nonunion development. This model provides a continuum between normal fracture healing and the development of nonunions.

## Introduction

Delayed or complete failure of fracture healing remains a problematic complication during fracture healing, with general incidences ranging between 5 and 10% [1, 2] and up to date, the pathophysiologic mechanisms for delayed fracture healing are not completely elucidated [3].

During the last decade(s), in vivo research in rodents has resulted in a wide range of different animal models for fracture healing and compromised healing resulting in delayed union and nonunion development [4, 5]. These models have to be standardized and need to mimic the human clinical situation as close as possible. Previous studies have investigated closed induction [6–8] of the fracture and open surgical procedures [5, 9], with differences in osteotomy size (critical sized segmental defects vs non-critically sized), and different fixation techniques as bridging plates [10-12], intramedullary nails [7, 13, 14] and external fixators [15, 16].

Several factors have been shown to influence bony healing such as the biomechanical environment (interfragmentary instability), inadequate blood supply [17] as well as the defect size [18, 19]. Availability of knockout mice and senescence altered mice allows a broad spectrum of molecular biology-based investigations [20] into developmental biological issues such as bone and cartilage formation [9, 12, 17] in combination with these different healing models.

Another key player in adequate fracture healing is the periosteum and integrity of the periosteum must be retained to achieve a successful fracture healing [21]. The periosteum consists of a thin, well-vascularized and innervated layer along the cortex of the bone and is primarily composed of osteogenic and fibroblastic cells [22]. Especially during the soft callus formation, the periosteum has a major influence on fracture repair as the periosteal progenitor cells will differentiate into osteoblasts and, mainly, chondrocytes [23, 24]. Consequently, in the present study, we hypothesize that periosteal cauterization would induce a significant and substantial delay in the bone healing process in mice. The aim of the current project is to describe and characterize the delayed healing process so that this developed novel model can be used for future biomechanical and molecular research to investigate the delayed bone healing process or its treatment.

## Materials and methods

### Animals and study design

A total of 87, 20–25 week old, skeletally mature, female, C57BL/6 mice (RCC Ltd, Füllingsdorf, Switzerland) were used in this study. Mice were housed socially in group cages with water and a standard maintenance diet (Provimi, Provimi Kliba AG, Kaiseraugst, Switzerland) ad libitum and with a 12-h day–night cycle. Before the surgical procedures, mice were randomly assigned to the control group or the periosteal cauterization group and each group of mice was equally subdivided into five sub-groups for different follow-up times (7, 14, 21, 28, and 42 days, see Table 1 for number of mice per group, analysis type and time of follow-up).

**Table 1:** Randomization of mice per study group and conducted analysis

Group	Days of follow-up				
	7 days	14 days	21 days	28 days	42 days
Control	8	10	10	9	8
Periosteal cauterization	8	9	8	8	9
Analysis	µCT X-ray	µCT X-ray histology	4-Point bending µCT X-ray histology	4-Point bending µCT X-ray histology	4-Point bending µCT X-ray histology

The ethical committee of the Canton of Grison, Switzerland approved the experimental set-up and all (surgical) procedures conducted in this study.

### Anaesthesia, analgesia, and surgical procedure

**Fig. 1:**
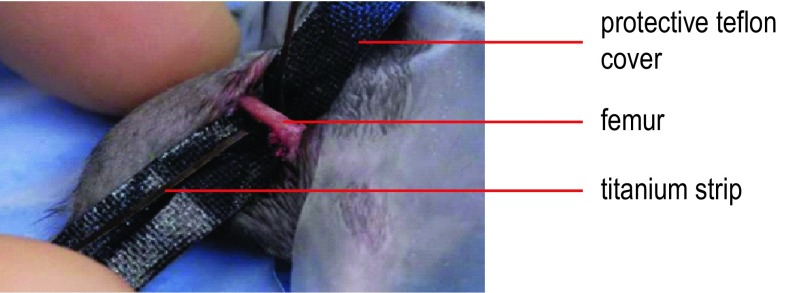
Cauterization of periosteum. During the surgical procedure, a titanium strip is placed circumferentially around the femur and subsequently attached to the cauterization device. A protective Teflon cover is placed under the femur and over surrounding soft tissues

General anaesthesia, analgesia and the surgical approach and postoperative pain treatment were carried out as previously described [12, 25]. Briefly, the mice were operated under general anaesthesia using isoflurane after obtaining pre-emptive analgesia consisting of buprenorphine (Temgesic), which was continued for 24 h every 8 h postoperatively. Additionally, mice received paracetamol per os for 5 days. In mice that were assigned to the periosteal cauterization group, a 0.8 mm thick titanium foil was pulled tight around the mid-shaft of the femur, held with forceps and an electrome was used to cauterize the periosteum circumferentially for 0.5 s with use of a protective Teflon cover around the other tissues (see Fig. 1). In all animals, a four-hole internal fixating plate (Titanium, 7.0 × 1.5 × 0.7 mm, MouseFix™, RISystem AG, Davos, Switzerland) [11] was placed on the lateral aspect of the femur and, after predrilling with a 0.33 mm drill bit, secured with four 2.0 mm angular stable screws (MouseFix™, RISystem AG, Davos, Switzerland). Following fixation, a 0.45 mm mid-diaphyseal femoral gap osteotomy was performed using a Gigli wire saw and irrigation with 0.9% NaCl. In the group with cautery, the osteotomy was placed in the middle of the periosteal injury resulting in a 0.25 mm-wide strip of injured periosteum on the proximal and distal side of the gap. To induce secondary healing, the screws were loosened half a turn to induce some degree of instability into the fixation [25]. Free weight bearing was allowed immediately after recovery from anaesthesia.

Animals were euthanized using CO_2_ following the different time periods of fracture healing as shown in Table 1 and both the right femur which underwent the osteotomy as the untouched left femur from each mouse were excised.

### Mechanical testing

In mice euthanized after 21, 28, and 42 days of healing, the plate was gently removed and both femora were immediately tested in non-destructive four-point-bending (ElectroForce 3220, Bose ESG, Eden Prairie, MN, USA). Femora were bent with the former plate position on the compression side at 2.1°/min to 4.5 N mm. The linear portion of the curve was used to calculate the bending stiffness. Each femur was tested three times. The healing femur stiffness was averaged and normalized by the contralateral intact femur stiffness. Bones from earlier time points (1, 7 and 14 days) were too fragile to test due to insufficient bone healing.

### Micro-computed tomography analysis

All bones were analysed by Micro-computed tomographic (µCT) imaging (µCT 40, Scanco Medical, Bassersdorf, Switzerland): after excision and gentle removal of the plates (time points 7 and 14 days) or mechanical testing (timepoints 21, 28 and 42 days) all osteotomized femora were fixed in 100% methanol. µCT was performed as described in previous studies [12, 25] to evaluate the fracture gap of all bones. Three-dimensional reconstructions with a special resolution/voxel size of 12 µm were made and based on a histogram of attenuation distribution, tissue was segmented into two types: woven bone (low mineralization, 14.5–36.0% of maximal gray value) and lamellar bone (high mineralization, > 36%).

**Fig. 2:**
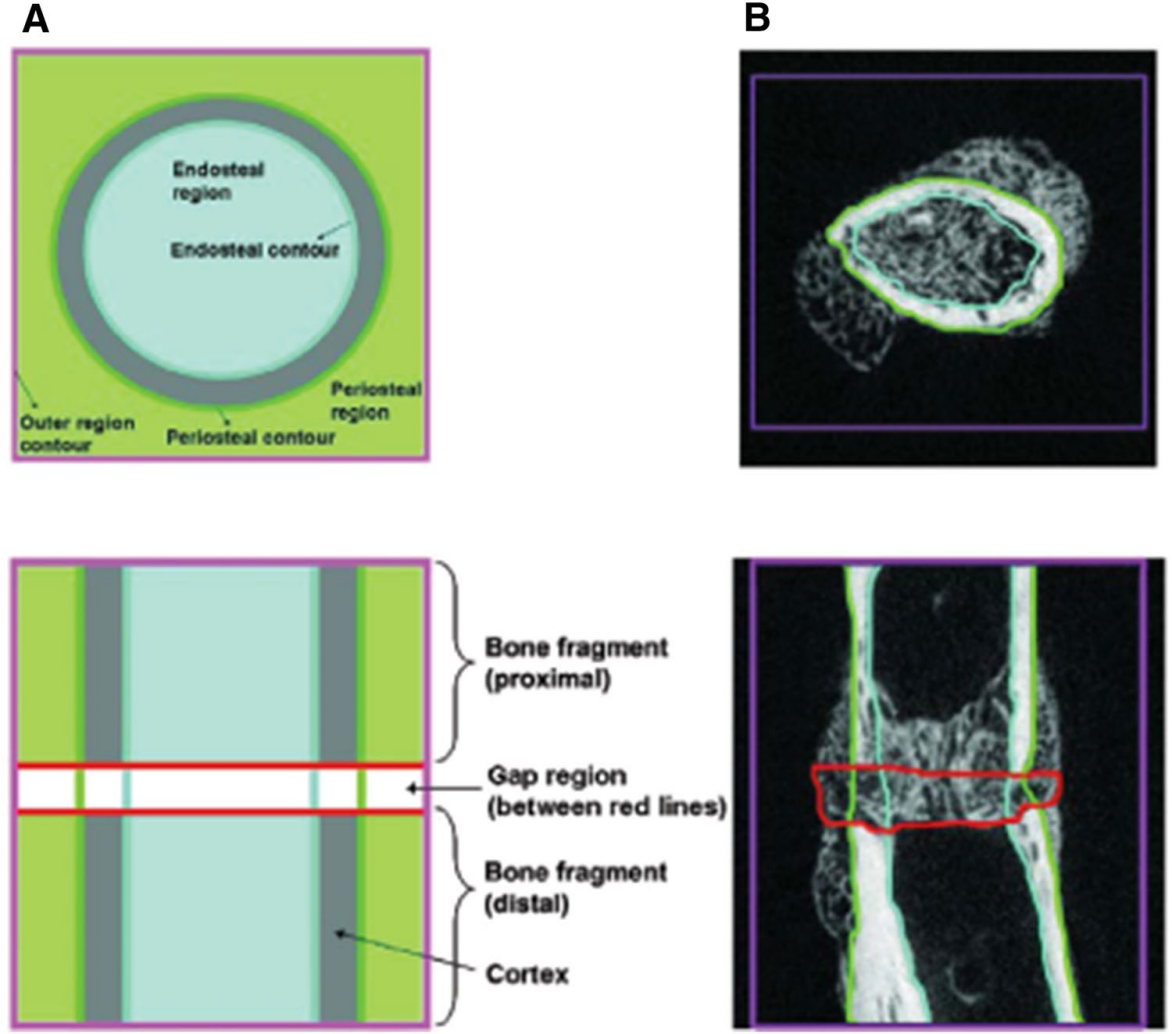
Definition of regions of interest during micro-CT analysis. Schematic representation of the four regions of interest: total region (TOT), periosteal region (PER), endosteal region (END) and the gap (GAP) between the fracture parts (**a**). The border of the complete investigated region (TOT) is the complete surrounding of the tissue between the most proximal and distal placed screws (purple borders). Green and light blue borders represent the periosteal and endosteal contours and regions of interest. The GAP region is marked by red lines at the osteotomy site. In **b**, previously mentioned borders are drawn in a representative micro-CT image. In both **a** and **b**, the upper image shows a transverse cross-sectional representation of the femur and the lower image a longitudinal representation

For precise quantitative analysis, different regions of interest (ROIs) were defined (see Fig. 2 for a schematic overview). The largest, total region of interest (TOT) included the entire scanned volume between the most proximal and distal placed screws. The periosteal region (PER) comprised any new bone tissue starting at the outer cortical boundary of the femora and extending radially outward, while the endosteal region (END) contained all newly formed bone within the medullary cavity, i.e. within the inner cortical boundary of both fragments. The actual fracture gap (GAP) was defined as the space between both fragments and its extension radially outward. The GAP region included only newly formed tissue, any bone fragments and original mid-diaphyseal cortex were excluded.

### Radiographic score

Blinded postoperative and post-mortem radiographs as well as cross-sectional CT images per sample of all timepoints were graded based on callus formation, rebridging of the cortices and callus remodelling using the radiographic scoring scale of Garrett et al*.* [26] (see Table 2). Radiographs provided the overview of fragment alignment and callus formation while 16 two-dimensional CT-cross-sections, equally spaced in the central part of the GAP region were assessed blindly by three medically trained investigators. Results are presented as median with maximum score.

**Table 2:** Radiographic-scoring scale according to Garrett et al. [26]

Score	Definition
0	No bridging, no callus formation
1	No bridging, initiation of a small amount callus
2	No bridging, obvious callus formation near fracture
3	No bridging, marked callus formation near and around fracture site
4	Rebridging of at least one of the cortices, marked callus formation near and around fracture site
5	Rebridging of at least one of the cortices, marked and complete callus formation around fracture site
6	Rebridging of both cortices, and/or some resolution of the callus
7	Clear rebridging of both cortices and resolution of the callus

Based on rebridgement of the cortices and acceleration of healing

### Histology

For histological analysis, femora of both groups after 14, 21, 28 and 42 days of fracture healing were decalcified (12.5% EDTA with 1.25% NaOH), embedded in paraffin and cut into 6 µm thick sections. Immunohistochemistry was performed for collagen II and collagen X to compare the time course for chondrocyte maturation and differentiation as described previously [25]. Sections were counterstained with haematoxylin and eosin to provide a clear overview of the images. Evaluation was performed qualitatively (Axioplan. Carl Zeiss AG, Feldbach, Switzerland) using transmitted light at 50 × magnification.

### Statistical analysis

Normal distribution of all subgroups was tested using Shapiro–Wilks test. An analysis of variance was performed with periosteal injury and healing time as factors in a full factorial general linear model using post-hoc Tukey correction. Differences at specific time points were tested with one-way ANOVA for “time” and independent *t* test for “treatment” using post-hoc Bonferroni correction (significance threshold *p* < 0.01). Inter-observer agreement was tested using Fleiss’ *κ* and afterwards, differences in radiographic scoring between groups were analysed by nonparametric Kruskal–Wallis test and Mann–Whitney *U* test. *p* values below 0.05 were considered statistically significant unless stated otherwise. Analysis was performed using GraphPad Prism 6 (GraphPad, San Diego, California, USA). Data in this paper are represented as mean values and standard error of the mean (SEM).

## Results

### Mechanical testing

**Fig. 3:**
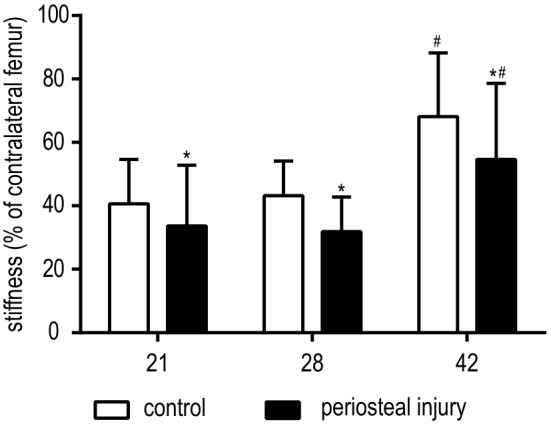
Biomechanical testing. Results of four-point bending stiffness after 21, 28 and 42 days of healing, results are presented as % stiffness compared with the contralateral (unfractured) femur. Control animals are shown in white bars, the periosteal cauterization group in black. Significance: *p < 0.05 when compared to control. ^#^*p* < 0.001 when compared to day 21 or 28

Bending stiffness assessed during four-point mechanical bending tests of healing bones at 21, 28 and 42 days post-surgery were significantly lower for the periosteal injury group when compared to control animals (all *p* < 0.05, Fig. 3). In both groups stiffness was significantly higher at 42 days of healing when compared to both previous time points (both *p* < 0.001).

### Micro-computed tomography analysis

**Fig. 4:**
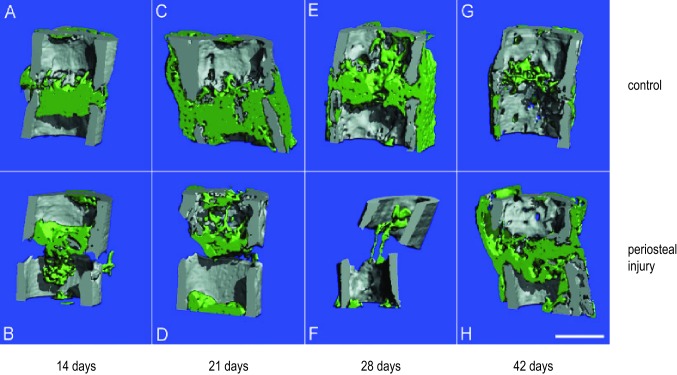
Reconstructed three-dimensional qualitative micro-CT images. Representative micro-CT images of femurs after 14 (**a**, **b**), 21 (**c**, **d**), 28 (**e**, **f**) and 42 (**g**, **h**) days of healing in the control group and periosteal injury group, respectively. Highly mineralized tissue is shown in gray, a lower degree of mineralization in green. Scale bar in **h** represents 1 mm and can be translated to the other panels

Reconstructed three-dimensional µCT images (Fig. 4) demonstrated that in the control group, fracture healing took place with immediate initial callus formation and subsequent resorption (day 28, Fig. 4e) and remodelling of the callus (day 42, Fig. 4g). In contrast, in the periosteal injury group no periosteal reactions were noticeable at the beginning (Fig. 4b, d, f) and callus formation was both delayed and reduced. By day 42 (Fig. 4h), the fracture healing process had started and an immature callus was observed in the periosteal cauterization group.

**Fig. 5:**
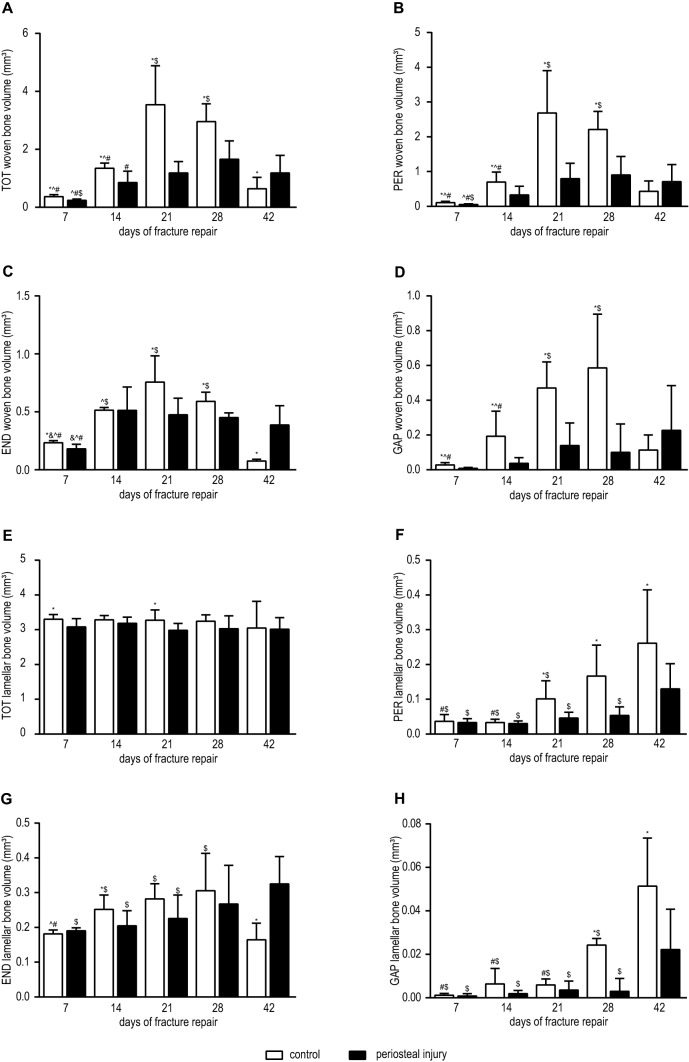
Woven and lamellar bone volumes in all four regions of interest. White bars represent control animals without periosteal cauterization, black bars mice with periosteal injury. *TOT* total region, *PER* periosteal region, *END* endosteal region, *GAP* osteotomy gap between proximal and distal part of the femur. **a**–**d** Show volumes of woven bone in the four different regions of interest, and **e**–**h** volumes of lamellar bone. *p < 0.05 when compared to periosteal injured mice at same time point. ^&^*p* < 0.05 when compared with 14 days of healing. ^^^*p* < 0.05 when compared with 21 days of healing. ^#^*p* < 0.05 when compared with 28 days of healing. ^$^*p* < 0.05 when compared with 42 days of healing

Quantitative µCT evaluation of the TOT region of interest showed that reduced volumes of woven bone are formed during the healing process in the periosteal injury group before day 42 (*p* < 0.05; Fig. 5a). In both groups, woven bone volumes changed over time. Mice in the control group showed a substantially steeper increase in woven bone volumes, especially between 14 and 21 days in comparison with animals that underwent periosteal cauterization. After 21 days, bone volumes in control animals reached a maximum, whereas woven bone volumes in the periosteal injury group increased at a lesser, steady rate until day 28 (*p* < 0.05 when compared with measured femora after 7 and 14 days of fracture healing). Thereafter, the volume of woven bone decreased in the control group until the end of the experiment after 42 days when compared with samples collected after 21 and 28 days of fracture healing (both *p* < 0.0001). In the periosteal injury group, the woven bone volumes remained elevated at 42 days of healing.

Woven bone volumes in the PER (Fig. 5b) and END (Fig. 5c) regions showed again a peak at 21 days of fracture healing (both *p* < 0.0001 when compared to 7 days), which afterwards subsequently decreased until the end of the experimental period at 42 days (both *p* < 0.0001 when compared with 21 days of healing). In the periosteal injury group, an increase was observed in woven bone volumes in the PER region between 7 and 21 days of healing, which afterwards stayed almost constant at a plateau level until the end of the experimental period (all *p* < 0.05 when compared to samples collected after 7 days of healing). A similar pattern of bone volumes was present in the END region during fracture healing in mice with periosteal injury. The peak in periosteal woven bone volume (Fig. 5b) in control animals was a twofold higher when compared with the periosteal injury group (*p* < 0.001) and to a lesser extent also in the endosteal region of interest (*p* < 0.05).

Periosteal injury suppressed callus growth in the GAP region with significantly less woven bone volumes between 7 and 28 days of fracture healing (*p* < 0.01 at every time point) and a maximum volume which is a two–threefold lower when compared with normal fracture healing in control mice (Fig. 5d).

Total lamellar bone volumes did not differ significantly between 7 and 42 days in control animals as well as mice with periosteal injury (Fig. 5e). Volumes of lamellar bone in the PER region of interest (Fig. 5f) increased significantly in both groups until the end of the experiment (both *p* < 0.0001); however, at the end of the experimental period, femora in the control group showed a twofold higher bone volume compared with samples after periosteal injury (*p* < 0.05). Lamellar bone volumes in the endosteal region (Fig. 5g) showed an increase in until postoperative day 28 in control mice (*p* < 0.05) and subsequent diminished volumes at 42 days (*p* < 0.001). In the periosteal injury group, the peak of lamellar bone volume was at the end of the experimental period at an increase of ~ 50% when compared with measurements taken after 7 days (*p* < 0.01). Finally, in the GAP region between the proximal and distal part of the femur, lamellar bone volumes increased by 50-fold in the control group (*p* < 0.001 when compared to day 7) and about 25-fold in mice with compromised healing (*p* < 0.01, *p* < 0.05 between both groups of animals).

### Radiographic score

**Fig. 6:**
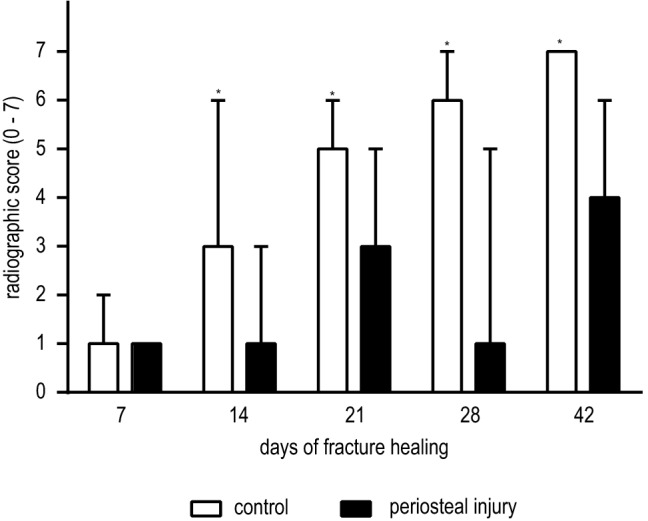
X-ray evaluation of fracture healing. Results of radiographic evaluation of healing at day 7, 14, 21, 28 and 42 after the femur osteotomy. Evaluation was performed by three independent researchers according to the scoring-scale by Garrett et al., with results presented as median with maximum score. White bars represent control mice, black bars show mice with periosteal cauterization. *p < 0.05

*κ* values for each of the three observer pairs were 0.49, 0.38 and 0.34, respectively. The overall inter-observer agreement was fair (0.40). In the control femora, the osteotomies healed progressively with lower variation among animals over time (Fig. 6). In the group with periosteal injury, a higher variability in healing progress was registered, especially after 28 days of healing. However, after 42 days, the radiographs demonstrated a step forward in healing with consistently higher scoring in these animals. Radiographic grading indicated consistent earlier and more advanced healing in control animals when compared to mice with periosteal injury starting at postoperative day 14 (*p* < 0.05) and until the end of the experimental period at day 42 where cortical bridging was not always attained in the periosteal injury group. The delay in score magnitude ranged between approximately 1 and 2 weeks.

### Histology

**Fig. 7:**
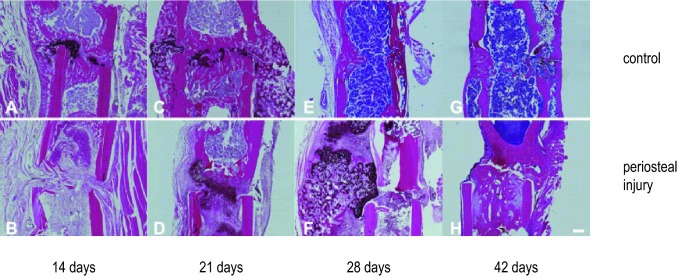
Collagen II immunohistochemistry. **a**, **c**, **e**, **g** Represent mid-sagittal femur histology slides stained for collagen II after 14, 21, 28 and 42 days, respectively, with hematoxylin and eosin counter staining. In **b**, **d**, **f**, **h** femurs of mice with periosteal injury are represented at the same time points. Images are made at a × 50 magnification, the scale bar represents a size of 200 µm

**Fig. 8:**
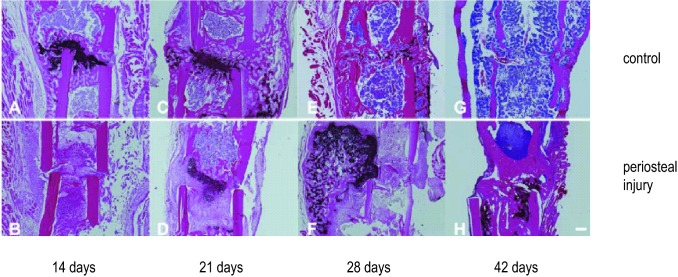
Collagen X immunohistochemistry. **a**, **c**, **e**, **g** Represent mid-sagittal femur histology slides stained for collagen X after 14, 21, 28 and 42 days, respectively, with hematoxylin and eosin counter staining. In **b**, **d**, **f**, **h** femurs of mice with periosteal injury are represented at the same time points. Images are made at a × 50 magnification, the scale bar represents a size of 200 µm

Histological results corroborated the quantitative outcome from the µCT analysis. Based on the expression of collagen II (Col II) and collagen X (Col X), on the formation and resorption of cartilaginous tissue in the gap and on the bridging between the fragments, the healing course was clearly prolonged in the group with periosteal injury. In femora of the control group, a more robust expression of Col II and Col X (respectively, in Figs. 7 and 8) was observed between postoperative days 14 and 28. After 14 days, the callus in the fracture gap consisted of woven bone in combination with cartilage which was mainly located in the centre of the gap region (Figs. 7a, 8a). At day 21 a more massive periosteal reaction was visible, whose cartilaginous portion was increasingly replaced by woven bone as evidenced by intense Col X staining (Figs. 7c, 8c). At day 28 (Figs. 7e, 8e) both cortices were bridged with woven bone and remodelling had already started, noticeable by the advanced stage of callus resorption around the periosteum and in the endosteal cavity. In the group with periosteal injury, woven bone formation and the amount of cartilage were delayed, as demonstrated by the expression of Col II and Col X. After 14 days (Figs. 7b, 8b) only connective tissue and no callus was visible in the fracture gap. At day 21 (Figs. 7d, 8d) Col II and Col X were detected representing the amount of cartilage located within the cortical boundaries of the two fragments. On day 28 after the surgical procedure (Figs. 7f, 8f), an extensive cartilaginous callus was formed and included some mild amounts of woven bone; reflecting a delayed reaction to a persistent instability of the fracture fixation and mechanically inadequate stabilization with fibrous tissue and cartilage.

## Discussion

The aim of the current project was to develop an in vivo murine model for delayed union development, as an intermediary between normal fracture healing and the development of nonunions, for future possibilities in biochemical and molecular research to investigate enhanced and deficient bone healing processes. Results demonstrated that the fracture gap obtained after a standardized osteotomy reduced with semi-rigid internal plate-screw osteosynthesis and combined with periosteal injury prolonged the healing period for 7–14 days, with callus formation volumes after 42 days of fracture healing which were comparable with callus after 21–28 days in the control group. In contrast, in the control group without periosteal cauterization resorption of the fracture callus via remodelling processes was well advanced with restoration of the femur diameter and reconstruction of the medullary canal. Therefore, this model of delayed fracture healing provides an ideal intermediate between normal fracture healing and nonunion development, whereas larger sized osteotomies would result in critical segmental defects resulting in nonunion development without the ability to assess the enhanced healing capabilities of future bone-healing strategies.

Quantitative results from the µCT analysis showed that as a consequence of the periosteal injury, the typical healing response was inhibited with the amount of woven bone in and mostly around the osteotomy was significantly reduced. Bridging of the proximal and distal fragments with mineralized lamellar bone was delayed accordingly. Radiographic analysis showed similar patterns in fracture repair with a 1–2 week delay in the periosteal injury group. Immunohistochemical evaluation on formation, maturation, and hypertrophy of chondrocytes using Col II and Col X markers also demonstrated a shift in the fracture repair response as shown in Figs. 7 and 8. In the periosteal injury group, the normal healing cascade was delayed and prolonged with fibrous connective tissue and cartilage still present in the gap region, chondrocytes which just started to hypertrophy, limited presence of woven bone and no complete bridging of the cortices evident. As a result, postponed healing delayed functionality as bending stiffness increased over time for both the control group as the mice with periosteal injury. Stiffness at the end of the experimental period was significantly higher in control animals when compared with periosteal injured mice due to a larger callus supporting the osteotomy and a higher degree of bone mineralization. As other isoforms, i.e., collagen I are mainly found in mature bone, these were not investigated in the present study. Collagen III, which is found in scar tissue and connective tissue, next to the blood vessel walls, has been reported to regulate osteoblastogenesis [27, 28]. However, the most pronounced delayed union and nonunions in our model are observed between day 28 and 42 whereas collagen III is mainly found between the 5th and 20th postoperative day and additionally does not significantly affect the callus volume in the early stages of fracture repair [27], therefor making collagen III a less reliable marker in our current investigation.

The electro cauterization procedure performed in this study destroyed the integrity of the periosteum on the proximal and distal side of the osteotomy gap. Disruption of the periosteum leads to a markedly impaired blood supply [22, 29-31] and subsequent to a reduced release and proliferation of various cell types and to a reduced capacity to form bone and cartilage [17, 32]. The critical role for the periosteum explains the obtained results in this study that in the periosteal injured group of mice during the first 2 weeks of fracture healing neither chondrocytes nor osteoblast-specific cells were migrating to the osteotomy gap and only fibrous tissue did develop.

Extensive reviews have been published on in vivo models of fracture healing and delayed union and nonunion development in rodents [33–35]. A wide range of different models have been created to study biomechanical and biomolecular processes during fracture repair and compromised fracture healing.

Standardized closed fracture models have been developed which induce fractures by three of four-point bending [8] or using a blunt guillotine combined with a dropping weight [6]. In these models, the fracture will represent a more realistic situation as is seen clinically with a better containment of the fracture hematoma. As compared to our newly developed model, a disadvantage is, as this is not a model of compromised fracture healing, that a relatively low number of delayed unions/nonunions which will occur decreasing the usability for studying the biomolecular and biomechanical processes during delayed fracture repair. Also, since there is relatively thin soft tissue coverage of the tibia, its influence on fracture healing and possible interplays between different tissues is difficult to assess in this model [34].

A range of different intramedullary fixation methods are presented in literature used in closed fracture models [7] and in open [5, 9] surgical procedures. Minimally invasive methods used are accompanied by a lack of rotational and axial stability and as a result have a high risk of dislocation [7], making them not useful in standardized delayed union research. More adequate models using intramedullary pins are accompanied by locking nails [8, 13] or compression screws [14] making it possible to use segmental defects for studying compromised fracture healing. However, all intramedullary fixation techniques severely damage the medullary canal, making it impossible to study the different endosteal processes during healing of the bone [34].

Until now, delayed union studies in mice and rats have been conducted using external fixators [15, 16], intramedullary pins [5, 9, 36] or no fixation at all [17]. The use of unilateral or circular external fixation devices ensures minimal disturbances of the fracture/osteotomy location during healing but also in subsequent analysis. However, the relatively high weight of the fixators and possible excessive micromovement when using unilateral fixators will increase the unpredictability of the obtained results [34, 35].

Plate-screw osteosynthesis with locking plates and screws [11] as used in the current study is designed for minimal periosteal contact and can as such be used to investigate influence of periosteal modification on fracture healing and keeping the advantages of an intact medullary canal when compared with the intramedullary fixation methods. Reproducible results have been obtained in the current study and previously [10, 12].

Although mice are not an exact model for human fracture healing, since rodents lack a Haversian system but use comparable resorption cavities for bone remodeling [4, 37], a major advantage of murine models is the reduced time (and costs) necessary for experiments since the healing process under normal circumstances takes around 3 weeks until there is no detectable motion between the fracture parts [33, 38]. In the current investigation, we had better controlled biomedical conditions as compared to other fixation techniques [39–41], and advantages over models which use tibial fracture healing as the straight longitudinal axis of the femur makes standardized fracture stabilization and accuracy of biomechanical testing easier and more reproducible. Recently, titanium-covered PEEK (polyether ether ketone) was developed and used which mimic the titanium surface of human osteosynthesis materials [10]. From an ethical point of view, every animal can then be monitored multiple times and during longer periods and without the need for euthanasia prior to collecting data, which is in compliance with the principles of reduction, replacement and refinement in lab animal experiments.

Mice also have a broad range of possibilities for usage of gene-targeted (knockout/knockin) animals, which enables molecular mechanistic studies on bone repair [42] and different existing models are present e.g. in research aimed at osteoporosis based fracture healing in senescence accelerated mice [43]. The periosteal injury model discussed in this current study has been used in the recently published study on the influence of nitric oxide (NO) deficiency on delayed bone healing and nonunion development [12]. In short, in this study, knockout mice deficient for nitric oxide synthase (a key enzyme necessary for NO production) showed nonunion development when compared with normal wild type control animals, after a femur osteotomy combined with periosteal cauterization, as used in the current study. At the end of the experimental period after 42 days of fracture healing, the deficient animals showed no presence of callus formation and bone volumes which were between two- and fivefold lower when compared with mice in the control situation.

When interpreting the obtained results, some limitations need to be considered. In the periosteal injury group, some longer time points for the follow-up period would be needed to assess if the healing process continues and subsequently results in remodelling of the callus as is shown in the control group. With these extra time points, the final delay in healing could be assessed. Next to this, we only investigate one factor leading to the delay in fracture healing and control other confounding factors such as the biomechanical environment. In this model for delayed fracture healing this is a strength resulting in reproducible data; however, since bone healing in general is a multifactorial process, further research is needed into other influential factors. A final minor point of attention is the fair interobserver agreement which was reached in the radiographic analysis; however, this limited value underscores the micro-computed tomography results which show comparable and significant quantified results of bone and callus formation.

In conclusion, a moderate fracture gap produced by osteotomy and fixated by flexible plate-screw osteosynthesis in combination with additional periosteal injury induced by electro cauterization leads to a delayed union development in a murine in vivo model. The periosteal injury induced a delay of healing time of 1–2 weeks compared to control samples, visible as callus formation and gap bridging and the presence of collagen expression within the gap region. The observed delay is considered to be clinically relevant since normalized by averaged healing time in mice (4 weeks) [41] and humans (16–20 weeks), it can be extrapolated that a delay of about 1–2 weeks in mice would correspond to delayed healing in humans by around 4–6 weeks. In the future, this mouse model with periosteal injury can be used to evaluate basic research questions regarding involvement of certain pathways or genes or to develop diagnostic tools and treatment options, in a model that provides a continuum between normal fracture healing and the development of nonunions.
